# Prediction of Communication Impairment in Children With Bilateral Cerebral Palsy Using Multivariate Lesion- and Connectome-Based Approaches: Protocol for a Multicenter Prospective Cohort Study

**DOI:** 10.3389/fnhum.2022.788037

**Published:** 2022-01-31

**Authors:** Jie Hu, Jingjing Zhang, Yanli Yang, Ting Liang, Tingting Huang, Cheng He, Fuqin Wang, Heng Liu, Tijiang Zhang

**Affiliations:** ^1^Department of Radiology, Medical Imaging Center of Guizhou Province, The Affiliated Hospital of Zunyi Medical University, Zunyi, China; ^2^Department of Diagnostic Radiology, The First Affiliated Hospital of Xi’an Jiaotong University, Xi’an, China; ^3^Department of Radiology, The First Affiliated Hospital of Henan University of TCM, Zhengzhou, China; ^4^Department of Radiology, Chongqing University Central Hospital, Chongqing, China

**Keywords:** cerebral palsy, children, magnetic resonance imaging, prediction, communication

## Abstract

**Background:**

Bilateral cerebral palsy (BCP) is the most common type of CP in children and is often accompanied by different degrees of communication impairment. Several studies have attempted to identify children at high risk for communication impairment. However, most prediction factors are qualitative and subjective and may be influenced by rater bias. Individualized objective diagnostic and/or prediction methods are still lacking, and an effective method is urgently needed to guide clinical diagnosis and treatment. The aim of this study is to develop and validate an objective, individual-based model for the prediction of communication impairment in children with BCP by the time they enter school.

**Methods:**

A multicenter prospective cohort study will be conducted in four Chinese hospitals. A total of 178 children with BCP will undergo advanced brain magnetic resonance imaging (MRI) at baseline (corrected age, before the age of 2 years). At school entry, communication performance will be assessed by a communication function classification system (CFCS). Three-quarters of children with BCP will be allocated as a training cohort, whereas the remaining children will be allocated as a test cohort. Multivariate lesion- and connectome-based approaches, which have shown good predictive ability of language performance in stroke patients, will be applied to extract features from MR images for each child with BCP. Multiple machine learning models using extracted features to predict communication impairment for each child with BCP will be constructed using data from the training cohort and externally validated using data from the test cohort. Prediction accuracy across models in the test cohort will be statistically compared.

**Discussion:**

The findings of the study may lead to the development of several translational tools that can individually predict communication impairment in children newly diagnosed with BCP to ensure that these children receive early, targeted therapeutic intervention before they begin school.

**Trial registration:**

The study has been registered with the Chinese Clinical Trial Registry (ChiCTR2100049497).

## Introduction

Cerebral palsy (CP) is the most common physical disability in childhood and occurs in 2–3 per 1,000 live births (Patel et al., 2020). Recently, the majority of individuals with CP achieve a life expectancy close to that of the general population. Clinical and research interests now focus on improving the ability of children with CP to perform activities of daily living and socially interact. Communication plays an integral role in people’s daily activities and social participation, yet only a few studies have examined the prevalence and features of communication impairment in children with CP (Mei et al., 2016; Kristoffersson et al., 2020). Communication (receptive and/or expressive language) impairments are common comorbidities in patients with CP, with a prevalence ranging from 46 to 78% (Coleman et al., 2015, 2016; Mei et al., 2016; Kristoffersson et al., 2020). Communication problems result in poor prospects of engagement and social participation in children with CP across a range of activities, including self-development, social functioning, and learning (Pennington et al., 2019; Vaillant et al., 2020). Particularly by the time these children enter school, communication skills greatly impact school readiness, which affects later social and academic success as well as economic and health outcomes (High, 2008).

Language development from birth to 2 years, when brain plasticity is at its greatest, presents multiple opportunities for intervention (Chorna et al., 2017). During this period, language perception (receptive) and production (expressive) are accompanied by the development of cortical areas responsible for improvements in working memory and pattern recognition in the first year and the multiplication of associations between different cortical processing areas in the second year (McMahon et al., 2012). Social interactions and interventions leveraging cortical development and cortical associations between language and motor systems at this critical time represent a promising role in improving long-term language outcomes and social functions (Goldstein et al., 2003; McMahon et al., 2012; Lipscombe et al., 2016; Chorna et al., 2017). Therefore, the key to interventions in these individuals lies in the importance of the early identification of children with CP at high risk for communication impairments (Coleman et al., 2015).

Several studies (Coleman et al., 2015; Hustad et al., 2018; Pennington et al., 2020; Tan et al., 2020) have attempted to explore demographic variables, early clinical characteristics, and environmental factors in predicting communication ability in children with CP at school entry. Studies have demonstrated that communication, cognition, and motor performance at an early age are critical for predicting future functional communication in children with CP (Coleman et al., 2015; Hustad et al., 2018; Pennington et al., 2020; Tan et al., 2020). However, most standardized tests require behavioral repertoires that exceed the capabilities of young children with CP, which makes these measures too difficult to use to assess these children reliably (Geytenbeek et al., 2010; Nordberg, 2018). Other non-standardized tests, such as parent-completed measures and grade-level classification systems, are unlikely to easily and adequately capture the scope of information necessary to track impairment-related changes (Geytenbeek et al., 2010; Potter, 2016). All these limitations make these methods less accurate in identifying or predicting children with CP at high risk for communication impairment.

In recent decades, progress in magnetic resonance imaging (MRI) techniques has increased the number of opportunities to search for more precise and objective biomarkers in various pediatric neurological diseases; additionally, MRI has been recommended and widely used for diagnosis, prognosis, treatment monitoring and research in CP (Graham et al., 2016; Novak et al., 2017). Three studies (Geytenbeek et al., 2015; Coleman et al., 2016; Choi et al., 2017) used qualitative categorical descriptions from MRI to investigate the characteristics of brain abnormalities associated with communication performance in children with CP. All these studies found that communication is related to the type of brain lesion on MRI (qualitative measurement); specifically, more severe periventricular white matter lesions (PWMLs) are associated with worse communication performance for the patient. Moreover, some researchers (Coleman et al., 2016; Laporta-Hoyos et al., 2018) used a semiquantitative MRI scale that provides scores by evaluating the extent of brain injury—assigning higher scores for increased lesion involvement—to assess the severity of brain damage in children with CP. The results demonstrated that children with CP with more severe lesions in the presumed language pathway regions (the left frontal, left temporal, left thalamus, left posterior internal capsule, left caudate and lenticular nuclei) had poorer overall communication skills and lower Communication and Symbolic Behavior Scales Developmental Profile (CSBS-DP) total scores (Coleman et al., 2016). All these results indicate that MRI has potential value in predicting communication impairment in children with CP. However, the above studies had a cross-sectional design, and variables reported as risk factors were qualitative or semiquantitative. Thus, an early quantitative and individualized prediction model for communication outcomes based on patient characteristics and MRI parameters is still lacking.

Multivariate lesion- and connectome-based approaches (Gleichgerrcht et al., 2017) are emerging techniques that provide an objective and quantifiable way to statistically evaluate the relationship between whole-brain structure connections and behavioral function. Notably, a prediction model constructed from this approach had high efficacy in the prediction of language deficits in post-stroke patients (Yourganov et al., 2016) and motor outcomes in neonates with arterial ischemic stroke (Al Harrach et al., 2021). Therefore, this study aims to develop individual prediction models based on multivariate lesion- and connectome-based approaches at an early age to predict communication ability in children with CP by the time they enter school. Among all types of CP, bilateral cerebral palsy (BCP) is the most common subtype, comprising 64.7% of all cases (Colver et al., 2014). Furthermore, different neuroimaging findings have revealed different patterns of insult to the brain in CP; recent research has noted that white matter lesions are the most common findings in imaging, accounting for 49.1% of all cases, and the severity of PWMLs has been verified to be linked with communication performance in children with CP (Geytenbeek et al., 2015; Coleman et al., 2016; Choi et al., 2017). Thus, we will also limit study participants to those with BCP and PWMLs (the most common subtype and neuroimaging finding in children with CP) to reduce the heterogeneity within the study population to improve the accuracy of prediction models. The results of this study may lead to the development of several translational tools that can be used to make individualized predictions of communication impairments in children newly diagnosed with BCP and PWMLs to ensure that these children receive early, targeted therapeutic intervention before they begin school.

## Aims

### Primary Aim

The aim of this study is to develop and validate an individual-based model that can be used to predict the communication impairment of children with BCP and PWMLs at school entry based on advanced brain MRI at an early age (before 2 years).

### Secondary Aims

(1) To establish a practical method for multivariate lesion- and connectome-based approaches of children with BCP and PWMLs.
(2) To identify injuries to cortical regions and connectomes associated with communication impairment in children with BCP with PWMLs.

## Materials and Methods

### Study Design

This study will be implemented as a multicenter prospective cohort study at four centers. This study has been registered with the Chinese Clinical Trial Registry (ChiCTR2100049497) and will be reported in accordance with the Standard Protocol Items: Recommendations for clinical Trials (SPIRIT) (Chan et al., 2013).

#### Study Setting

The study will take place at the Affiliated Hospital of Zunyi Medical University (No. 149, Dalian Road, Huichuan District, Zunyi City, Guizhou Province, China), the First Affiliated Hospital of Xi’an Jiaotong University (No. 277 Yanta West Road, Xi’an, Shaanxi, China), the First Affiliated Hospital of Henan University of Chinese Medicine (No. 19 Renmin Road, Zhengzhou, Henan, China), and Chongqing University Central Hospital (No. 1 Jiankang Road, Yuzhong District, Chongqing, China). The study will begin in December 2021 and is expected to be completed in June 2025.

#### Ethics and Dissemination

All participating centers approved this study protocol. Full ethics committee approval was obtained from the Institutional Review Board of The Affiliated Hospital of Zunyi Medical University (KLL-2021-108). A signed, informed consent form will be obtained from the legal parents/guardians of each participant. Participating subjects will receive a summary of the results, including clinical phenotype descriptions, imaging reports, and results of the behavioral assessments they have completed. The results of the present study will be submitted to peer-reviewed journals and/or reported at relevant conferences. Subjects will be able to withdraw from the study at any time without explanation. They will not suffer any penalty from the staff, nor will any repercussions be evident in their care due to this decision.

### Participants

Some investigators have noted that predictors of communication difficulties may differ for the subgroup of children with CP in early life (Pennington et al., 2020). Therefore, we will limit our study participants to those with BCP (associated with more serious complications and a more common prevalence of communication problems) (Geytenbeek et al., 2015) to find reliable prognostic markers for this population. Brain MRI-based classification systems have been widely recommended and used for classifying different types of brain lesions in children with CP, and different neuroimaging methods may reveal the different pathogenic patterns responsible for CP (Ditchfield, 2017; Himmelmann et al., 2017). Among all the types of lesions, PWMLs are the most common neuroimaging finding in this population (Himmelmann and Uvebrant, 2011; Coleman et al., 2016; Himmelmann et al., 2017), and the severity of PWMLs has been shown to be associated with the severity of communication impairment (Geytenbeek et al., 2015; Coleman et al., 2016; Choi et al., 2017). Thus, we will also constrain neuroimaging to children with BCP and PWMLs to reduce the heterogeneity within our study population.

#### Inclusion Criteria

Children meeting the following criteria will be considered for enrollment in this study: (I) confirmed diagnosis of BCP by pediatric neurologists (Novak et al., 2017), (II) age 6 months to 2 years at the time of recruitment, (III) PWMLs as described on the MRI imaging report, and (IV) willingness by participants and parents to participate in the study.

#### Exclusion Criteria

Children will be excluded if they have any one of the following criteria: (I) blindness, severe visual impairment or hearing impairment (given the requirements of some language assessment modules); (II) other diseases (i.e., hereditary disease, cancer, severe infectious disease, severe heart disease, or progressive central nervous system diseases); (III) MRI artifacts affecting any further image processing analysis; (IV) insufficient cooperation to participate in the communication assessment; and (VI) a primary language other than Chinese among the family members.

#### Withdrawal Criteria

Subjects will be withdrawn from this study at the discretion of the researchers if there are safety concerns or if the children are unable to complete follow-up examinations.

#### Sample Size

There are no directly available data to assess the relationship between connectome-based features and clinical assessments of children with BCP to predict their communication ability at school entry. The sample size was calculated according to a previous study (Coleman et al., 2016). In that study of children with CP with a total sample size of *n* = 131, the number of children with communication abnormalities was 62 (47.0%). Multiple linear regression showed that lesion type and five semiquantitative indices that reflected the severity of brain lesions were related to the communication assessment score. However, the efficient size f2 cannot be directly calculated because the R2 and residual variance in the multiple regression model were not reported in the study (Coleman et al., 2016). Geytenbeek et al. (2015) used multiple linear regression based on MRI measures to evaluate language comprehension in non-speaking children with severe CP and PWMLs; that study revealed that the R2 of the model was 0.36. Choi et al. (2017) also found significant negative relationships between the severity of PWMLs and expressive language and between the severity of PWMLs and receiving language performance. Although the above results indicate that the MRI measures could be used to adequately predict communication performance in children with CP, we still assumed a lower efficient size for the MRI measures (f2 as 0.10) to ensure a sufficient sample size for an accurate prediction of communication performance in children with CP. Based on previous studies (Geytenbeek et al., 2015; Coleman et al., 2016; Choi et al., 2017), the number of predictors was set as 6. If α = 0.05, 1-β = 0.80, then the study requires enrollment of 143 participants according to the power calculation by G*Power, version 3.1.9 (Faul et al., 2009). According to previous follow-up experience and given a maximum anticipated dropout rate of 20%, a total sample size of 178 children with BCP and PWMLs will be needed.

### Study Procedures

**FIGURE 1:**
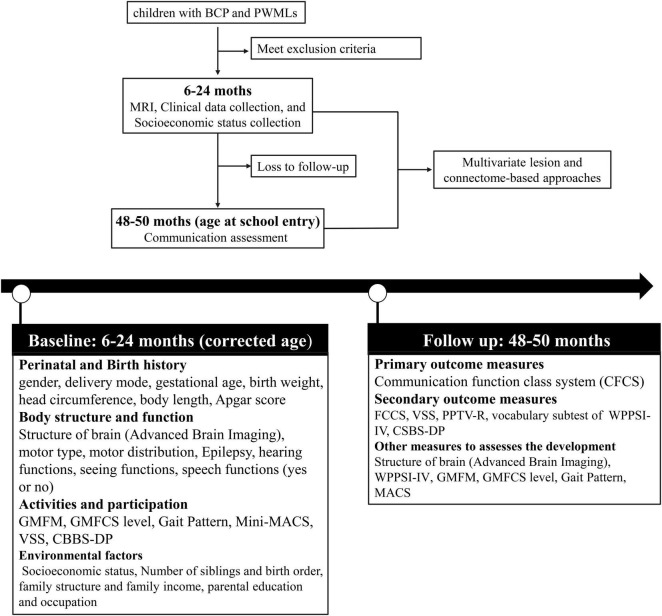
Flow chart of the study protocol. BCP, Bilateral Cerebral Palsy; PWML, Periventricular White Matter Lesion; MRI, Magnetic Resonance Imaging; GMFM, Gross Motor Function Measure; GMFCS, Gross Motor Function Classification System; MACS, Manual Ability Classification System; VSS, Viking Speech Scale; CBBS-DP, Communication and Symbolic Behavior Scales Developmental Profile; FCCS, Function Communication Classification System; PPTV-R, Peabody Picture Vocabulary Test-Revised; WPPSI-IV, Wechsler Preschool and Primary Scale of Intelligence.

The study procedures are shown in Figure 1. Following the inclusion and exclusion criteria, eligible participants (children with BCP and PWMLs) will be entered from corrected ages of 6 to 24 months. Participants will be assessed for diagnostic criteria, differential diagnosis and comorbidities by a pediatrician or child neurologist. The participants will undergo at least 1 advance brain MRI examination according to a standardized protocol. A variety of clinical data and data pertaining to socioeconomic status will be collected. Neurobehavioral development will be assessed by experienced physiotherapists blinded to the MRI findings and related medical information (such as other examination results relevant to the content of the current assessment) at each center.

At the age of 48–60 months, the participants will be invited back to the hospital for a follow-up assessment. Experienced speech and language therapists (SLTs) blinded to the medical history will assess each child’s communication ability using multiple classification systems and scales. The primary outcome measure for this study is the communication function classification system (CFCS) rating. Secondary outcome measures include ratings from the Functional Communication Classification System (FCCS), Viking Speech Scale (VSS), Peabody Picture Vocabulary Test-Revised (PPTV-R), verb comprehension subtest of the Wechsler Preschool and Primary Scale of Intelligence (WPPSI-IV), and CSBS-DP Infant-Toddler Checklist. The questionnaire data (CSBS-DP) will be collected by trained researchers during a telephone survey for participants who are reluctant to return to the hospital for follow-up. Other measures, such as Gross Motor Function Classification (GMFCS), Manual Ability Classification System (MACS), WPPSI-IV, and advanced brain imaging, will also be used to evaluate the development of participants during the follow-up assessment.

### Blinding

The neuroradiologists involved in lesion delineation and MRI analysis will be blinded to other clinical information and assessment findings. The child neurologists and physiotherapists conducting the neurobehavioral assessment at baseline will be blinded to the MRI findings. Follow-up assessments will be implemented once the children are old enough for preschool by SLTs blinded to the children’s clinical history, baseline assessment and MRI findings.

### Clinical Data Collection

#### Demographic Data

Basic demographic and clinical information will be collected from families and health records. The following general information will be collected: age at MRI examination, sex, body weight, height, medical history, comorbidities, epilepsy, family makeup, seizure history, and medication. In addition to the above information, prenatal factors have also been shown to be related to brain injury and affect language development outcomes. Thus, birth history variables, including delivery mode, gestational age, birth weight, head circumference, body length, and Apgar score, will also be documented.

Furthermore, a systematic review (Vaillant et al., 2020) indicated that the factors in some domains of the International Classification of Functioning, Disability and Health – Children and Youth framework are relevant for language comprehension in children with CP. According to this concept, children’s motor type, motor distribution, hand function (MACS), gross motor function (GMFCS), hearing function, visual function, speech function, socioeconomic status, parental educational level, parental occupation, and birth order will be recorded.

#### Primary Outcome Measures

The aim of the present study is to predict communication performance in children with CP at school entry. This will be assessed using the CFCS, which contains 5 levels (I-V), with level I being the best functional and level V being the least functional level. Children will be classified into one of the 5 levels based on their performance in sending and receiving information with familiar and unfamiliar communication partners. The CFCS is the most widely used system designed to classify communication function in children with CP in daily life. To increase the accuracy of the assessment, this study will establish a standardized assessment process according to a previous study (Wang and Wei, 2017). In this process, (I) SLTs will question parents regarding different aspects of the child’s daily function and simultaneously observe children’s movements during the initial contact (understanding and discovering the ways in which the child usually communicates), (II) the child’s communication with the family will be observed and recorded, and (III) SLTs will further communicate with the child through play to allow the child to fully demonstrate his or her communication skills.

While the SLTs are conducting the on-site CFCS rating, a caregiver will be asked to read the CFCS-Chinese version and conduct a retrospective assessment of the child’s communication performance in daily life. Then, the caregiver will be asked to determine his or her child’s CFCS level by taking into account the communication performance status on site. If there are 2 caregivers in the field, the ratings will be made simultaneously without discussion, and the one who spends more time with the child will be marked as the first caregiver and the other one will be marked as the second caregiver. The SLT will explain the CFCS language but will not discuss the CFCS outcome with the parents. The final CFCS levels will be determined by professionals (SLTs) in close collaboration with parents. The CFCS has good reliability with high intraclass correlation coefficients across different language versions (Hidecker et al., 2011; Vander Zwart et al., 2016; Wang and Wei, 2017; Choi et al., 2018; Mutlu et al., 2018) and has been widely used in multiple CP registries (Coleman et al., 2015; Hustad et al., 2016; Mei et al., 2016; Vander Zwart et al., 2016; Wang and Wei, 2017; Mutlu et al., 2018) as a recommended, suitable tool for evaluating everyday communication in children with CP. The CFCS-Chinese version has also shown good reliability with intraclass correlation coefficients of 0.88 between an SLT and a caregiver and of 0.86 between the first and second caregivers for children with CP (Wang and Wei, 2017).

#### Secondary Outcome Measures

The secondary outcome measures include the levels/scores of the FCCS, VSS, PPTV-R, CSBS-DP, and the vocabulary subtest of WPPSI-IV for implementing a comprehensive communication assessment of children with CP. Table 1 describes the classification systems of the CFCS, FCCS, and VSS.

(1) FCCS: The FCCS is a 5-level system, with the highest level (level V) indicating the worst performance. The FCCS classifies children’s performance in sending communicative messages and considers their observable communication performance using speech, gestures, and/or augmentative and alternative communication. The reliability and validity of the FCCS for classifying the functional communication of children with CP aged 4 to 5 years have been previously tested (Barty et al., 2016).
(2) VSS: The VSS (Pennington et al., 2013) was developed to classify the speech production of children with CP. The VSS is a four-level rating scale used to classify speech intelligibility in children with CP aged 4 years and above when they are speaking to unfamiliar partners and strangers. It has substantial to almost perfect test-retest reliability (κ > 0.68) and moderate to substantial interrater reliability between pairings of professionals and/or parents (κ = 0.58–0.81) (Pennington et al., 2013).
(3) PPTV-R: The PPTV-R is often used to assess the receptive vocabulary abilities of people ranging in age from 2.5 to 40 years old, and the reliability and validity of the Chinese version have been previously tested (Sang and Miao, 1990). The test is composed of 175 items, and its test-retest reliability and split-half reliability are 0.94 and 0.99, respectively. One point is recorded each time a picture is correctly matched to the given word. The raw score is obtained by summing all the correct item scores.
(4) CSBS-DP: The CSBS-DP (Wetherby and Prizant, 2002) is a parent questionnaire containing 24 questions and investigates children’s expressive and receptive language skills; verb/non-verbal communication skills; and symbolic skills, such as participation in pretend play and appropriate use of objects. The total raw scores are converted to a standardized score, with mean = 100 and standard deviation (SD) = 15 among typically developing children (Wetherby and Prizant, 2002). Scores below 80 imply developmental delay of communication and referral for further language and speech assessment. The test–retest reliability of the CSBS-DP for the composite and total scores ranged from 0.79 to 0.88 (Wetherby and Prizant, 2002), and the reliability and validity of the Chinese version were previously tested (Lin et al., 2015).
(5) WPPSI-IV: The Chinese version of the WPPSI (Zhang, 2009) is divided into two groups of tests: group 1 for children aged 2.5 years to 3 years and 11 months and group 2 for children aged 4 years and 0 months to 6 years and 11 months. The WPPSI-IV comprises 3 subtests (verbal comprehension, working memory and visuospatial ability) for group 1 but 5 subtests (verbal comprehension, working memory, visuospatial ability, fluid intelligence and processing speed) for group 2. The verbal comprehension test includes three subtests: similarity, comprehension, vocabulary, and a supplemental subtest—common sense. Verbal comprehension index scores have a mean = 100 and standard deviation = 15. If children with CP are unable to complete the verb comprehension subtest due to fine motor impairment, language impairment or reduced intellectual ability, a composite score will be calculated.

**TABLE 1:** Descriptors for levels of the CFCS, FCCS, and VSS.

Level	CFCS	FCCS	VSS
I	Effective sender/receiver with familiar/unfamiliar partners	Effective communicator in most situations	No speech motor disorder indicated
II	Effective, but slower-paced sender and/or receiver with familiar and/or unfamiliar partners	Effective communicator in most situations but may need help	Speech motor disorder indicated but is usually understandable to unfamiliar listeners
III	Effective sender and receiver with familiar partners; not effective with unfamiliar partners	An effective communicator in some situations	Speech motor disorder indicated and is not typically understandable to unfamiliar listeners out of context
IV	Inconsistent sender and/or receiver with familiar partners; not effective with unfamiliar partners	Assistance required in most situations, especially with unfamiliar partners	No understandable speech
V	Seldom effective sender/receiver with familiar partners; not effective with unfamiliar partners	Communicates unintentionally using movements and behavior	–

*CFCS, Communication Function Classification System; FCCS, Functional Communication Classification System; VSS, Viking Speech Scale.*

### Clinical Data Collection

#### Acquisition of MR Images

Brain MR images will be acquired using 3.0-T GE scanners with 8-channel head coils at baseline. All participants will be scanned during natural sleep, as their sleep times will be adjusted based on the experimental schedule, and earplugs will be used to reduce the scanning noise from the MRI chamber. In cases where the child cannot fall asleep to comply with the MRI examination, sedation will be administered after obtaining parental or guardian consent. The potential risks of sedation will be fully explained to the parents or guardians. Each subject’s vital signs will be monitored closely, and head motion will be limited by the placement of foam padding around the child’s head during the MRI examination. Three sequences will be acquired: 3-dimensional fast spoiled gradient-recalled echo (3D-FSPGR) T_1_-weighted imaging (T_1_WI), T_2_-fluid attenuated inversion recovery (T_2_-FLAIR) imaging and diffusion tensor imaging (DTI). Image acquisition parameters of 3D-FSPGR, T1WI, T2-FLAIR, and DTI will be held consistent across sites to reduce the variability of imaging data. The detail scan parameters of the MRI sequences are shown in Table 2.

**TABLE 2:** Scan parameters of the MRI sequences.

Variable	T_2_-FLAIR	3D-FSPGR T_1_WI	DTI
Repetition time (ms)	7500	7.8	12500
Echo time (ms)	145	3.0	86.8
Number of diffusion gradient directions	NA	NA	64
*b* values (s/mm^2^)	NA	NA	0,1000
Slice thickness (mm)	3	1.0	2.5
Gap (mm)	1.5	0	0
Field of view (mm^2^)	240 × 240	256 × 256	240 × 240
Matrix size	256 × 256	256 × 256	256 × 256

*T_2_-FLAIR, T_2_-fluid attenuated inversion recovery; 3D-FSPGR T_1_WI, 3-dimensional fast spoiled gradient-recalled echo T_1_ weighted imaging; DTI, diffusion tensor imaging; NA, not available.*

#### Lesion Masks

Periventricular white matter lesions will be characterized as areas of T_2_ hyperintensity with or without cystic degeneration. The lesion masks will be manually delineated on T_2_-FLAIR images using ITK-snap software (Yushkevich et al., 2006) by two neuroradiologists. Intrarater and interrater reliability in lesion segmentation will be evaluated using the Dice κ consistency test (Guo et al., 2017). If there is an evident discrepancy, a third neuroradiologist will be invited to make the final decision. Then, the T_2_-FLAIR image will be coregistered to the T_1_ image, and these parameters will be used to reslice the lesion mask into the native T_1_ space. The resliced lesion masks will be binarized using a 50% probability threshold. The alignment between the resliced lesion masks and the lesions in native T_2_ space will be visually inspected by two experienced neuroradiologists by comparing the overlay of the resliced lesion mask to the patient’s native T_1_ image to the overlay of the original lesion mask on the patient’s native T_2_-FLAIR image. Cases of misalignment will be manually corrected directly in the normalized lesion mask using ITK-snap software.

#### Lesion (Voxel)-Based Analysis

Two complimentary approaches (lesion- and connectome-based) will be used to characterize brain damage. Both of them will use a brain atlas developed by Oishi et al. (2011) to divide the brain into 122 regions to reduce the feature dimensions. For lesion-based and connectome-based analysis, the brain atlas containing the parcelation information will be aligned to each individual’s native T_1_ images. First, the T_1_ target image of patients will be created by using a groupwise template creation method (Li et al., 2016). Then the patients T_1_ target image will be registered to the infant T_1_WI template provided by Oishi et al. (2011) in the standard space. Subsequently, these deformation parameters will be inverted and applied on the brain atlas in the standard space to obtain the brain atlas in the local patient target space and individual’s native T_1_ space. The unified segmentation function of SPM12 (Ashburner and Friston, 2005) will be used to obtain the probabilistic gray and white matter maps from T_1_ images. These probabilistic gray maps (in native T_1_ space) will be further divided into regions of interest (ROIs) corresponding to the brain atlas. Then, lesion-based analysis will be computed as the proportion of lesioned voxels per ROI (each cortical and subcortical gray matter region corresponding to the brain atlas).

#### Connectome-Based Analysis

The structural brain connectome will be computed as the number of white matter streamlines that connect each pair of cortical and subcortical gray matter regions, and probabilistic tractography will be performed in individual’s diffusion space to construct the connectome. Thus, the tissue maps (including the ROI-segmented gray matte map) will be registered into an individual’s diffusion-weighted imaging (DWI) space. First, since tissue contrast is comparable between B0 and T_2_ images, the registered T_2_-FLAIR image (coregistered into the native T_1_ image) will be linearly normalized into the mean B0 image from the diffusion MRI sequence using the FMRIB Software Library (FSL) Linear Image Registration Tool (Greve and Fischl, 2009) (correlation ratio cost function, affine registration with 12 parameters and nearest neighbor interpolation). The transformation matrices will then be used to register the probabilistic maps of white and gray matter, and segmented cortical ROIs in native T_1_ space into DWI space.

Probabilistic tractography will be applied to evaluate the structural connectivity of pairwise cortical regions, which will be separately defined according to the brain atlas. Structural connectivity will be obtained by the probabilistic method of the FMRIB Diffusion Toolbox (FDT) (Behrens et al., 2007) for fiber tracking. FDT BEDPOST will be used to build the default distributions of diffusion parameters at each voxel. Probabilistic tractography will be obtained using FDT’s probtrackx (Behrens et al., 2007) (parameters: 5,000 individual streamlines drawn through the probability distributions on principle fiber direction, step length of 0.5 mm, 200 maximum steps, curvature threshold set at 0.2, and distance correction) since probabilistic tractography is theoretically capable of accommodating intravoxel fiber crossings. The probabilistic white matter map will be used as the waypoint mask (Yourganov et al., 2016; Gleichgerrcht et al., 2017). The cortical ROIs (corresponding to the brain atlas) in diffusion space will be used as seed regions for tractography. For each subject, the connectivity between cortical ROIs i and j will be calculated, defined as the number of streamlines arriving at j when i is seeded, averaged with the number of streamlines arriving at i when j is seeded. The calculation of the streamlines will be corrected based on the distance traveled (“distance correction” built into probtrackx). In addition, the number of streamlines between ROIs i and j will be divided by the sum of the volume of the two regions to eliminate the effects of the unequal sizes of the different cortical regions. These steps will be repeated iteratively to ensure that all cortical ROIs are used as seed regions. Once all iterations are completed, a connectivity matrix A will be constructed, where each entry Aij represents the connection weight between cortical ROIs i and j.

#### Harmonization

Multisite imaging studies are prone to technical variability across scans, including differences between the manufacturers, heterogeneity in the protocol and variations in the scanning parameters. Such unwanted variation can hinder the detection of important features and/or cause spurious findings. There is a need to remove the bias and non-biological variance caused by unwanted site effects. In this study, we will use ComBat (Fortin et al., 2017, 2018), a method that adjusts the mean value and variance between different groups by combining an empirical Bayes framework and the location/scale model, to harmonize the data. Studies have shown that the ComBat method performs well in preserving biological variability and removing the unwanted variation introduced by site for harmonizing the 3DT_1_ imaging (Fortin et al., 2018) and DTI (Fortin et al., 2017).

### Models for Predicting Communication Impairment

#### Preselection of Predictive Features

Since the number of streamlines between two cortical regions is averaged, the connectivity matrix is symmetrical with respect to the diagonal, and only the lower triangular matrix will be used for further analyses. For both lesion-based and connectome-based analyses, the feature set will include hundreds to thousands of feature vectors, and such high-dimensional data can cause the “curse of dimensionality.” To reduce the number of potential features introduced into the predictive model, a preselection procedure will be performed to exclude noisy or uninformative predictors from being fed into the prediction model, which can reduce the chance of model overfitting. First, for lesion-based analyses, only gray matter regions that are lesioned in more than 10% of the patients will be entered into the analysis. Second, for the links in the connectome matrix-based analyses, only those that are present in more than 75% of the patients will be entered into the analysis. After the above steps, each subject will have three feature sets: the lesion feature set, connectome feature set, and combination feature set.

#### Development and Validation of Prediction Models

**FIGURE 2:**
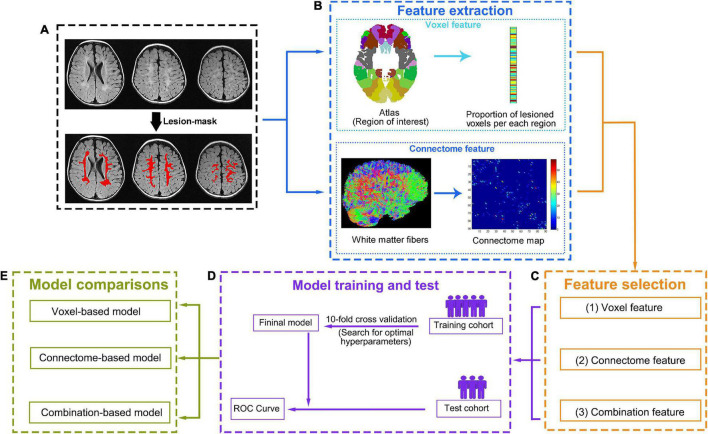
Data processing and analysis workflow. **(A)** First, the lesion will be manually defined on T_2_-FLAIR images. **(B)** Two complementary methods, one based on lesions and the other on the connectome, will be used to characterize brain damage. To compute lesion-based damage (top row), the brain atlas and each individual’s lesion mask will be aligned with each individual’s native T_1_ space. The gray matter regions will be divided into regions of interest (ROIs) according to the atlas. Then, lesion-based damage will be computed as the proportion of lesioned voxels per ROI based on the lesion mask. Connectome-based damage (bottom row) will be computed as the number of diffusion tensor imaging (DTI) tracts that connect each pair of ROIs (tractography for reconstructing tracts will be performed guided by the white matter probabilistic map). **(C)** For each individual, three feature sets (lesion features, connectome features, and combination features) will be generated from the corresponding features after feature selection. **(D)** For each feature set, a “training cohort” will be used to build an optimal model based on a 10-fold cross-validation, and the prediction performance of the optimal model will be tested with the “test cohort” using ROC curve. **(E)** Then, three optimal models will be generated based on different feature sets (the lesion feature set for voxel model, the connectome feature set for connectome model, and the combination feature set for combination model). The prediction performance of each model will be compared, and the best-performing model will be selected.

According to previously reported literature, for children with CP, the distribution of their CFCS grades is unbalanced and skewed. None or mild communication impairment (CFCS level I, II, III) in children with CP and PWMLs is more common than in those with severe communication impairment (CFCS level IV, V) (Kristoffersson et al., 2020). Such an imbalance distribution will cause misprediction during the process of predictive model generation (Saethang et al., 2016). Thus, we defined individuals who communicate at CFCS level I (defined as “effective sender and receiver communication with unfamiliar and familiar partners”) as having an absence of communication impairment. Patients with CFCS level I will be classified into the absence of communication impairment group, and those with CFCS levels II, III, IV, and V will be classified into the communication impairment group. In this way, the CFCS grade will be converted to a binary indicator, and our study will focus on identifying whether children with BCP have any communication impairment and improving the efficacy of the predictive model. All the variables will be centered and standardized before being entered into each model, which will be created with machine learning algorithms. In machine learning, the no-free-lunch theorem states that there is no one model (or, more generically, machine learning tool) that will work best for every problem (Wolpert and Macready, 1997). Therefore, several classification algorithms with varying hyperparameters will be evaluated using support vector machine, random forest, logistic regression, and other methods to investigate which algorithm will outperform competing approaches across all settings. The workflow of data processing and communication prediction is outlined in Figure 2. The detailed model building and validation steps are as follows.

Images from four sites (four hospitals) will be randomly partitioned into four equal portions. Approximately three-quarters of these images will be used as a “training cohort,” and the remaining one-quarter of these images will be used as a “test cohort.” In the “training cohort,” the proportion of lesioned voxels in the ROI and the links of the connectome matrix that are not shown difference between absence of communication impairment group and communication impairment group will be excluded, where the significance level is defined as *P* < 0.05 without multiple comparison correction in the two-sample test. The selected features will be used to define models predicting presence or absence of communication impairment in children with BCP. For each prediction model, 10-fold cross-validation (CV) will be used to evaluate the performance. This means that the “training cohort” will be divided into a training (nine folds) and a validation dataset (one fold), leaving 9 of 10 participants in the training dataset and the remaining participants in the testing dataset. For the training cohort, the various hyperparameters within each algorithm will be used to determine the prediction model. Then, the model will be applied to the validation dataset to predict children with the presence or absence of communication impairment (predicted label). The same procedure will be repeated with each of the one-fold participants or the left-out participants in the leave-one-out procedure, serving as the validation dataset for 10 iterations. Thus, each subject will receive a predicted label, and the accuracy of predictive model will be evaluate by comparing predicted labels to true labels of BCP children. The CV step will be repeated 100 times with random cohort designation to select the model with the best prediction accuracy. The whole “training cohort” will be reused in its entirety to retrain the model based on the classification algorithm and hyperparameters of the prior selected model. Then, the retrained model will be used to evaluate the “test cohort,” and the communication performance of every subject of the “test cohort” will be predicted by the model. The final accuracy of the prediction model will be defined by comparing predicted labels to true labels of BCP children in the “test cohort,” The Receiver Operating Characteristic (ROC) curve and the area under curve (AUC) will be calculated to evaluate the predictive ability of built models. A two-sided Hotelling-Williams test (Steiger, 1980) will be used to determine whether the multimodality prediction model is more accurate than any of the single-modality prediction models. *P* values < 0.05 will be considered indicative of statistically significant differences across models. Given the possible effects of age on imaging measures, the “test cohort” will be divided into three groups: younger (6–12 months), middle-aged (12–18 months), and elderly (18–24 months years), according to their age at MRI scan. The optimal model will be applied to all subgroups to exam whether the prediction accuracy changes with age at MRI scan.

### Confidentiality and Data Management

The original data will be kept confidential throughout the study and preserved by The Affiliated Hospital of Zunyi Medical University, China. The electronic data will be gathered at each site and supervised by the two specially trained investigators. The data will be stored in a secure computer, and only research staff will have access to the data. Unless required by law, none of the above information will be divulged. Anonymized study data will be published for scientific purposes.

### Trial Status

This trial is currently recruiting participants.

## Discussion

This study protocol reports the procedures for a prospective, multicenter, longitudinal cohort study that will establish an individual-based model for the prediction of communication impairment in children with BCP and PWMLs at school entry.

This study will have some potential strengths. Previous studies (Geytenbeek et al., 2015; Coleman et al., 2016; Choi et al., 2017; Laporta-Hoyos et al., 2018) have demonstrated that the type and severity of brain injury shown on MRI scans is associated with communication impairment. However, all these studies were retrospective in design and were performed to identify risk factors for communication impairment, and an individualized prediction model for communication is still lacking. Moreover, wide ranges of communication performance in the specified type or degree of injuries suggest the limited value of predicting communication outcomes based on quantitative and semiqualitative parameters (Geytenbeek et al., 2015; Coleman et al., 2016; Laporta-Hoyos et al., 2018). In this study, a large representative sample of children with BCP will be recruited, and the data will be collected prospectively using standardized neuroimaging protocols and outcome measures. An individual-based prediction model for communication ability will be developed based on quantitative MRI parameters. This model can provide information that can contribute to the determination of the appropriate time for intervention and guide decision-making. In addition, only a few articles (Geytenbeek et al., 2015; Coleman et al., 2016; Choi et al., 2017) have reported brain-communication relationships in children with CP, and neuroimaging characteristics identified as risk factors in these studies are quantitative and/or semiqualitative parameters. Hence, the neuroanatomic basis for communication impairment remains unclear (Vaillant et al., 2020). We will use voxel-based analyses to examine which regions are typically associated with communication impairment. Connectome-based analyses, a novel approach for identifying key connections for neurological function, will also be used to provide unique insights into communication deficits. In this light, it can be foreseen that the abovementioned methods would greatly help in the understanding of the neural basis of communication impairment in these children.

With the growing number of multisite neuroimaging studies, there is a great challenge in handling non-biological variance due to site-level factors, such as differences in MRI scanners and/or acquisition protocols. Such unwanted sources of variability and bias may mask true associations of interest and/or generate spurious findings. In this multisite neuroimaging study, we will use ComBat (Fortin et al., 2017, 2018), a well-established methodology for controlling unwanted variation induced by scanner and/or acquisition protocol differences, to remove site effects. Moreover, due to difficulties in obtaining neuroimaging data in children with CP (accompanied by multiple complications), our hospital will develop an individualized examination procedure for these children and extend it to other participating sites to ensure the feasibility of the process. During this period, translation of specialist knowledge and dissemination of research results into clinical practice will begin immediately via site investigators. The university affiliations across this study will provide opportunities to integrate research fronts and introduce this collaborative effort into undergraduate education. Knowledge translation will also target parents and/or guardians of children with CP to improve their quality of life.

This study will have several limitations. Given the multidimensional nature of communication, attempting to reduce communication skills to a single linear scale will be fraught with the problems of overlooking impairment-related data and important, informative developments (Potter, 2016; Nordberg, 2018). Therefore, the CFCS may not be able to capture the full scope of information necessary to evaluate cross-sectional performance and track the longitudinal development of communication ability. However, quantitative communication tests in the Chinese version for children with CP are scarce. The CFCS has been translated into different languages (Hidecker et al., 2011; Vander Zwart et al., 2016; Wang and Wei, 2017; Choi et al., 2018; Mutlu et al., 2018) and has often been used in multiple CP registries (Coleman et al., 2015; Hustad et al., 2016; Mei et al., 2016; Vander Zwart et al., 2016; Wang and Wei, 2017; Mutlu et al., 2018) worldwide. It shows good reliability and validity across different cultures and languages (including the Chinese version) and is quick and user friendly, making it ideally suited for multicenter trials. Thus, we will still consider CFCS as the primary outcome measure, and a search for precise scales in the Chinese version that enable quantitative evaluation of communication ability for these children will be continued. Functional MRI has great potential for characterizing typical development and detecting abnormalities early; however, it will not be included in our present study because of the poor ability of infants to tolerate MRI scanning (furthermore, after conventional and diffusion sequences, the evaluation time would be too long to include MRI examination). Finally, it should be noted that our study population is limited to children with BCP and PWMLs to reduce the heterogeneity in constructing more accurate predictive models. Thus, the prediction models constructed by this protocol will likely not be generalizable to other CP subtypes or BCP with other neuroimaging findings.

## Ethics Statement

The studies involving human participants were reviewed and approved by the Institutional Review Board of The Affiliated Hospital of Zunyi Medical University (KLL-2021-108). All participating centers approved this study protocol. Written informed consent form will be obtained from the legal parents/guardians of all participants.

## Author Contributions

JH, HL, and TZ designed and conceptualized the study and developed the first version of the manuscript. YY, TL, TH, and CH will provide assistance with the data acquisition protocol and methodology. JZ and FW helped to perform the statistical analyses and revised the manuscript for important intellectual content. HL carried out the design and critically revised the final version of the manuscript. All authors contributed to the article and approved the submitted version.

## Conflict of Interest

The authors declare that the research was conducted in the absence of any commercial or financial relationships that could be construed as a potential conflict of interest.

## Publisher’s Note

All claims expressed in this article are solely those of the authors and do not necessarily represent those of their affiliated organizations, or those of the publisher, the editors and the reviewers. Any product that may be evaluated in this article, or claim that may be made by its manufacturer, is not guaranteed or endorsed by the publisher.
